# LINC01116 accelerates nasopharyngeal carcinoma progression based on its enhancement on MYC transcription activity

**DOI:** 10.1002/cam4.2624

**Published:** 2019-11-08

**Authors:** Haijie Xing, Hongxia Sun, Weiluo Du

**Affiliations:** ^1^ Department of Otorhinolaryngology, Head and Neck Surgery University of Chinese Academy of Sciences Shenzhen Hospital Shenzhen China; ^2^ Department of Otorhinolaryngology, Head and Neck Surgery Affiliated Xinhua Hospital Hainan Medical College Haikou China; ^3^ Wuhan Medical Science Research Institute Wuhan China; ^4^ Affiliated Xinhua Hospital Hainan Medical College Haikou China

**Keywords:** LINC01116, MYC, nasopharyngeal carcinoma, tumorigenesis

## Abstract

Long noncoding RNAs (lncRNAs) have been demonstrated to be frequently involved in the development of cancers, whereas only a few of them was investigated in nasopharyngeal carcinoma (NPC). Here, we found that LINC01116 was highly expressed in NPC cell lines, and inhibition of LINC01116 notably restrained cell viability, proliferation, and migration in NPC cells. Besides, we unveiled that LINC01116 was mainly distributed in the cytoplasm of NPC cells. Surprisingly, the cytoplasmic LINC01116 could directly interact with the 5′UTR of MYC mRNA, whereas such interaction had no influence on MYC mRNA expression, but facilitated MYC mRNA translation so as to enhance MYC protein level in NPC cells. Moreover, LINC01116 per se had no impact on the transcription of MYC targets but affected their expression through MYC‐dependent manner. Furthermore, MYC overexpression offset the suppression of LINC01116 silence on NPC development. In turn, we discovered that MYC could also serve as the transcriptional activator of LINC01116 in NPC cells. By and large, our findings elucidated a LINC01116/MYC feedback loop in accelerating the tumorigenesis of NPC, revealing a promising target to establish novel biomarkers for NPC patients.

## Graphical abstract

LINC01116 contributes to cell proliferation and migration in nasopharyngeal carcinoma. Cytoplasmic LINC01116 interacts with MYC mRNA to stimulate MYC protein and therefore enhance MYC transcriptional activity in nasopharyngeal carcinoma cells. LINC01116 is transactivated by MYC protein in turn. The contribution of LINC01116 to nasopharyngeal carcinoma progression is mediated by MYC.

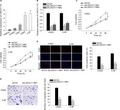

## 1 INTRODUCTION

Nasopharyngeal carcinoma (NPC) is a highly aggressive malignancy which usually occurs in South Asia, North Africa, and Southern China.^1^ NPC is possessed with quite different features compared to other head and neck cancers in several aspects, such as risk factors, pathogenesis, clinical behaviors, and therapeutic strategies.^2^ Similar to many other human cancers, the development of NPC is also a multistep process involving oncogene activation and tumor suppressor silencing.^3^, ^4^, ^5^ Despite extensive discoveries on genetic and epigenetic alterations in NPC,^6^ the understanding of the pathogenesis and progression of NPC remain unclear. Hence, it is important to figure out the precise mechanism underlying NPC development for the development of effective therapeutic strategies for NPC.

Recently, dysregulated LncRNAs have been largely recognized as crucial contributors in tumorigenesis of diverse human cancers,^7^ mainly due to their transcriptional and posttranscriptional regulation on gene expressions.^8^ Also, some lncRNAs have been proved to be implicated in NPC, such as n326322,^9^ HOTAIR,^10^ AFAP1‐AS1,^3^ and so on. Long intergenic nonprotein coding RNA 1116 (LINC01116) is a newly recognized lncRNA that locates in 2q31.1. Previously, studies demonstrated that LINC01116 is a carcinogene in several cancers.^11^, ^12^ However, its role in NPC has never been investigated yet.

In this study, we aimed to explore the role and potential mechanism of LINC01116 in NPC development.

## 2 MATERIALS AND METHODS

### 2.1 Cell culture

Human nasal epithelial cell (HNEpC), human embryonic kidney cell (HEK‐293T), and the NPC cells (HONE1, CNE1, CNE2, and 5‐8F) were all purchased from the Shanghai Cell Bank of the Chinese Academy of Sciences (China). All cells, except that HEK‐293T cells were cultured in Dulbecco's Modified Eagle Medium (DMEM; Gibco), were maintained in RPMI 1640 medium (Thermo Fisher Scientific) containing 10% fetal bovine serum (FBS; Thermo Fisher Scientific) and 1% antibiotics (penicillin/streptomycin; HyClone). Cell culture was conducted at 37°C in 5% CO_2_, along with the change of medium every three days.

### 2.2 Cell transfection

The shRNAs against LINC01116 (shLINC01116#1 and shLINC01116#2) or MYC (shMYC) and their corresponding NC (shCtrl), which were obtained from Genechem, were separately transfected into CNE2 or 5‐8F cells. Moreover, the pcDNA3.1 vectors (Genechem) containing LINC01116 or MYC was, respectively, used to enhance the expression of LINC01116 or MYC, whereas the empty vector served as negative control. Cell transfection with Lipotransfectamine 3000 (Thermo Fisher Scientific) lasted 48 hours.

### 2.3 Quantitative real‐time polymerase chain reaction (QRT‐PCR)

TRIzol reagent from Invitrogen was utilized to isolate total RNAs from cultured NPC cells. Reverse transcription of RNA into cDNA was achieved by RevertAid First Strand cDNA Synthesis Kit (Thermo Fisher Scientific). SYBR^®^ Premix Ex Taq™ II from Takara and Taqman Universal Master Mix II (Life Technologies Corporation) were applied on ABI PRISM 7300 Sequence Detection system (Applied Biosystems) for qRT‐PCR analyses. The 2^−ΔΔCt^ method was utilized to calculate the relative expression level of target gene in the experiment group and the control group.

### 2.4 Cell proliferation assays

Cell Counting Kit‐8 (CCK‐8; Sigma‐Aldrich) was applied for detecting proliferative ability of cells in accordance with instructions. Cells were placed into 96‐well plates (5 × 10^3^ cells in each well) at 37°C all night, and then treated with various transfection plasmids for 48 hours. Later on, 10 μL of CCK‐8 reagent was used to incubate cells for another 1 hour. The optical density (OD) value at 450 nm was monitored by a microplate reader. For EdU staining, the transfected NPC cells were subjected to EdU Alexa Fluor™ 555 staining (Thermo Fisher Scientific) as per the protocol. Nuclei were stained with DAPI. All experiments were run in triplicate.

### 2.5 Cell migration assay

Transwell migration assay (Corning) was utilized to assess migratory capacity of indicated NPC cells following the standard method. The cells were first planted into 12‐well plates with the density of 1 × 10^6^ cells/well. The serum‐free culture medium was used in the upper chamber and medium with 10% FBS in the lower chamber. Cells were cultured under 5% CO_2_ at 37°C for 72 hours, fixed in 70% ethanol for 10 minutes and stained with 0.5% crystal violet. Migrating cells were observed under an inverted microscope.

### 2.6 Nuclear‐cytoplasmic fractionation

CNE2 and 5‐8F cells were rinsed in pre‐chilled PBS solution and then cultured in 0.1% NP40 with protease inhibitor cocktail (Roche) and 10 mmol/L of Ribonucleoside Vanadyl Complex (New England BioLabs). Following centrifugation for 2 minutes, cell supernatant was reaped as cell cytoplasm, the remaining pellet was collected as the cell nuclei after washing in PBS.

### 2.7 RNA pull‐down assay

In vitro biotin‐labeled LINC01116 and its antisense RNA were obtained by transcription with the biotin RNA labeling mix (Roche) and T7 RNA polymerase (Roche) treated with RNase‐free DNase I (Promega). Following purification with RNeasy Mini Kit (QIAGEN), the biotinylated RNA was treated with cell lysates and finally pulled down by streptavidin agarose beads (Sigma‐Aldrich). The bound RNAs were collected for qRT‐PCR analysis.

### 2.8 Western blot assay

Cells lysed in RIPA buffer (Beyotime) were subjected to BCA protein assay kit II (BIO‐RAD). After separation by 10% SDS‐PAGE gel, protein samples were transferred onto nitrocellulose membranes (Invitrogen) and incubated with rabbit‐anti‐human MYC (ab9106; Abcam) and rabbit‐anti‐human GAPDH (ab9485; Abcam) antibodies overnight at 4°C. Horseradish peroxidase (HRP)‐conjugated secondary antibodies were subsequently used to incubate samples for 1 hour. The final blot signals on the protein samples were observed by ECL reagents (Millipore).

### 2.9 Luciferase reporter assay

HEK‐293T cells were put into the 24‐well plates for 24 hours of culturing. Artificially synthesized pGL3‐CDK4 promoter, pGL3‐CCND2 promoter, pGL3‐BMI1 promoter, and pGL3‐LINC01116 promoter were acquired commercially from RiboBio. The aforementioned reporter vectors were separately co‐transfected with indicated transfection plasmids into cells using Lipotransfectamine3000. Luciferase intensity was tested at 48 hours after transfection by Dual Luciferase Reporter Assay System (Promega).

### 2.10 Chromatin immunoprecipitation (ChIP)

To prepare ChIP assay, CNE2 and 5‐8F cells were cultivated in formaldehyde for 10 minutes to acquire DNA‐protein cross‐links. Afterward, lysates were sonicated to obtain DNA fragments of 200‐500 base pairs in length, followed by immunoprecipitation with anti‐c‐MYC and anti‐IgG as control. At length, the precipitated chromatin DNA was retrieved and analyzed with qRT‐PCR method, whereas the amplified DNA was further identified via agarose gel electrophoresis (AGE).

### 2.11 Statistical analyses

Experimental data of at least three independent samples were analyzed by GraphPad Prism 6.0 Software (GraphPad Inc) or SPSS 22.0 system (SPSS, Inc). Both Student's *t* test and one‐way ANOVA were applied for difference comparison statistically with the significant level of *P* < .05.

## 3 RESULTS

### 3.1 LINC01116 promotes NPC cell proliferation and migration

**Figure 1:**
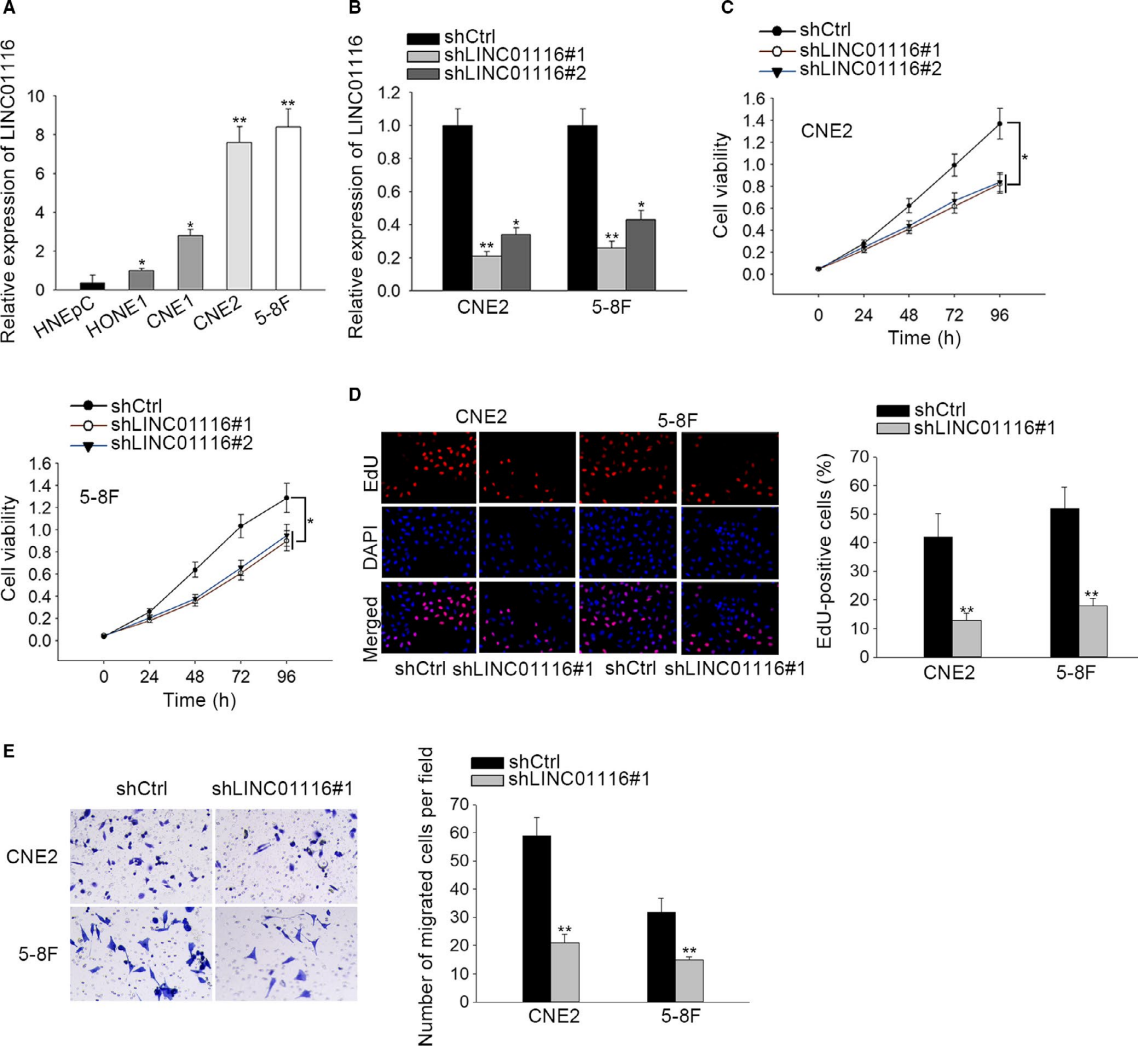
Inhibition of LINC01116 suppressed cell proliferation and migration in NPC cells. A, qRT‐PCR result of LINC01116 expression in NPC cell lines and the normal HNEpC cells. B, qRT‐PCR result of LINC01116 expression in CNE2 and 5‐8F cells under the transfection of shCtrl or two shRNAs against LINC01116. C, CCK‐8 assay was performed to evaluate cell viability in above cells. D, Cell proliferation was examined by EdU assay. E, Transwell assay for the assessment of cell migration in NPC cells with or without LINC01116 inhibition. *P < .05, **P < .01

To understand the role of LINC01116 in the development of NPC, we first detected its expression pattern in NPC cell lines and normal controls. Consequently, it was uncovered that the expression of LINC01116 was notably enhanced in NPC cells compared to that in the human nasal epithelial cell line HNEpc (Figure 1A). Subsequently, loss‐of‐function assays were conducted in CNE2 and 5‐8F cells which expressed relatively higher level of LINC01116. As proved by qRT‐PCR, the expression level of LINC01116 was overtly silenced in both CNE2 and 5‐8F cells responding to the transfection of shLINC01116#1 or shLINC01116#2 (Figure 1B). In addition, we revealed that the viability of NPC cells was markedly confined under LINC01116 inhibition, whereas the shLINC01116#1‐transfected cells showed a better knockdown efficiency (Figure 1C). Moreover, it turned out that depletion of LINC01116 led to restrained proliferative ability and migratory capacity in both CNE2 and 5‐8F cells (Figure 1D,E). On the contrary, gain of LINC01116 function resulted in strengthened viability, proliferative ability, and migratory capacity in HONE1 and CNE1 cells (Figure S1). Taken together, LINC01116 serves a tumor facilitator in NPC.

### 3.2 LINC01116 interacts with myc mRNA in the cytoplasm of NPC cells

**Figure 2:**
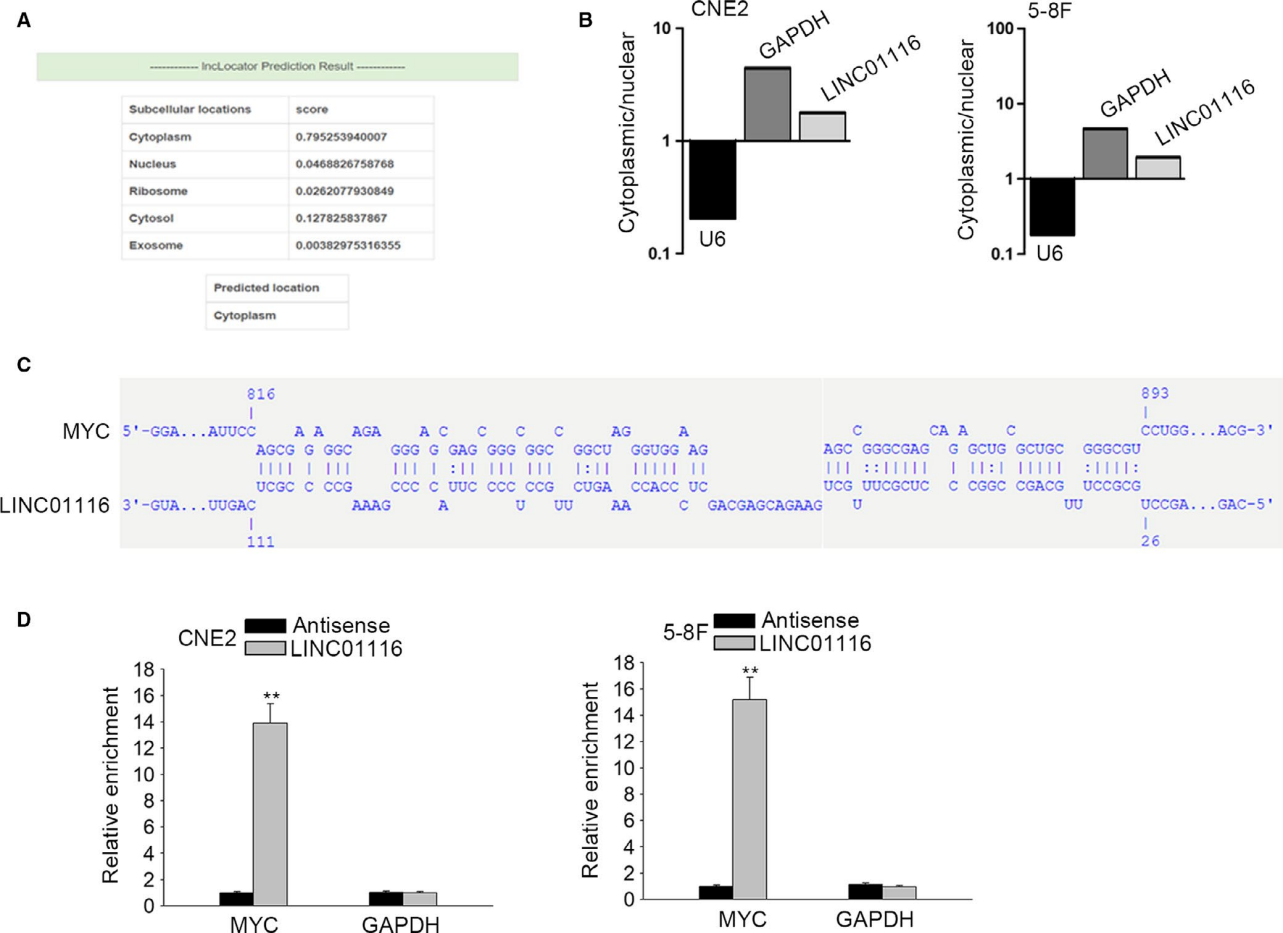
LINC01116 mainly located in the cytoplasm of NPC cells and interacted with the 5ʹUTR of MYC mRNA. A, LINC01116 was predicted by lncLocator as a cytoplasmic lncRNA. B, Subcellular fractionation plus qRT‐PCR validated that LINC01116 was mainly distributed in the cytoplasm of NPC cells. C, The online IntaRNA tool suggested that LINC01116 could bind to the 5'UTR of MYC mRNA. D, The interaction between LINC01116 and MYC mRNA in CNE2 and 5‐8F cells was confirmed by RNA pull down assay. **P < .01

Given that the function of lncRNAs varies according to their subcellular localization,^13^ we wondered where in which part of NPC cells LINC01116 located in. As predicted by lncLocator (http://www.csbio.sjtu.edu.cn/bioinf/lncLocator/), LINC01116 was mainly distributed in the cytoplasm (Figure 2A). Meanwhile, subcellular separation followed by qRT‐PCR indicated an apparent result that LINC01116 was concentrated mainly in the cytoplasm of NPC cells (Figure 2B). Previously, a recent report demonstrated that lncRNAs could modulate mRNA translation through interacting with the 5ʹ untranslated region (5′UTR) region of such mRNA.^14^ Here, we predicted that there was a potential interaction between LINC01116 and MYC 5ʹUTR through applying the online tool IntaRNA (http://rna.informatik.uni-freiburg.de/IntaRNA/Input.jsp) (Figure 2C). Furthermore, RNA pull down assay unveiled that MYC mRNA was mostly harvested by LINC01116 but not its antisense in both CNE2 and 5‐8F cells (Figure 2D). Jointly, these data uncovered that cytoplasmic LINC01116 interacts with MYC mRNA in NPC cells.

### 3.3 LINC01116 modulates myc activity through upregulating MYC protein

**Figure 3:**
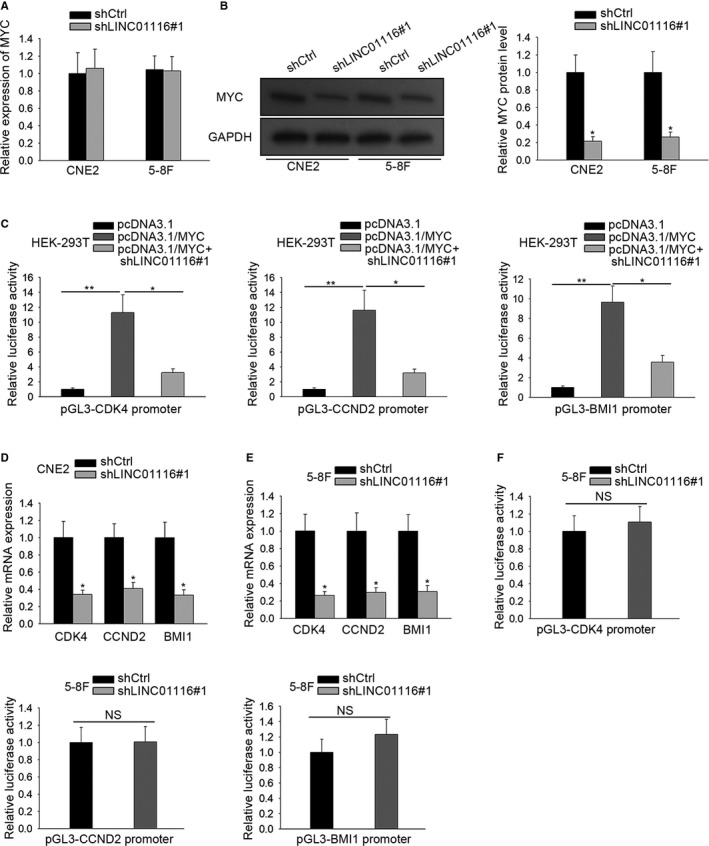
LINC01116 enhanced MYC protein level so as to promote the transcription of MYC targets. A,B, The impact of LINC01116 on the mRNA and protein levels of MYC was estimated by qRT‐PCR (A) and western blot (B). C, Changes on the transcription of three MYC targets (CDK4, CCND2, and BMI1) in the context of MYC overexpression or together with LINC01116 suppression were evaluated by luciferase reporter assays. D‐E, qRT‐PCR results of the expression of CDK4, CCND2, and BMI1 in LINC01116‐silenced NPC cells. F, The influence of LINC01116 per se on the transcription of CDK4, CCND2, and BMI1 in NPC cells was determined by luciferase reporter assay. *P < .05, **P < .01

Furtherly, we wondered the impact of LINC01116 on MYC. First of all, it was demonstrated that LINC01116 had no influence on the mRNA expression of MYC (Figure 3A). Surprisingly, knockdown of LINC01116 gave rise to obvious reduction on MYC protein in both CNE2 and 5‐8F cells (Figure 3B). More importantly, we observed that MYC protein was reduced in LINC01116‐silenced NPC cells due to hindered interaction between LINC01116 and MYC mRNA (Figure S2A,B); conversely, such interactivity was encouraged and therefore MYC protein expression elevated in HONE1 and CNE1 cells under LINC01116 overexpression (Figure S2C,D), suggesting LINC01116 affected MYC protein through its interaction with MYC mRNA. Recently, studies have proposed that antisense transcripts can facilitate the translation of selected mRNA in a cap‐dependent manner.^15^ In this situation, we supposed that LINC01116 interacted with MYC mRNA to facilitate MYC translation. Previously, researchers demonstrated that transient rapamycin treatment can suppress cap‐dependent translation.^16^ As anticipated, it was testified that the promotion effect of LINC01116 overexpression on MYC protein expression was abrogated in the presence of rapamycin (Figure S2E). In other words, LINC01116 boosts MYC protein through accelerating MYC translation.

Subsequently, we aimed to evaluate the influence of LINC01116 on c‐MYC activity. On this basis, we investigated whether silencing LINC01116 had an effect on the transcription of several identified c‐MYC targets, such as CDK4,^17^ CCND2, 18 and BMI1.^19^ As a result, the luciferase activities of all the three targets were enhanced in response to MYC overexpression, whereas such enhancement was then countervailed under the condition of LINC01116 suppression (Figure 3C). Importantly, the expression levels of CDK4, CCND2, and BMI1 were all decreased in LINC01116‐silenced CNE2 and 5‐8F cells (Figure 3D,E), although LINC01116 per se had no observable influence on their transcription (Figure 3F). On the basis of these results, we concluded that LINC01116 enhances MYC activity in NPC through enhancing c‐MYC.

### 3.4 Overexpression of MYC offset LINC01116 depletion‐controlled NPC development

**Figure 4:**
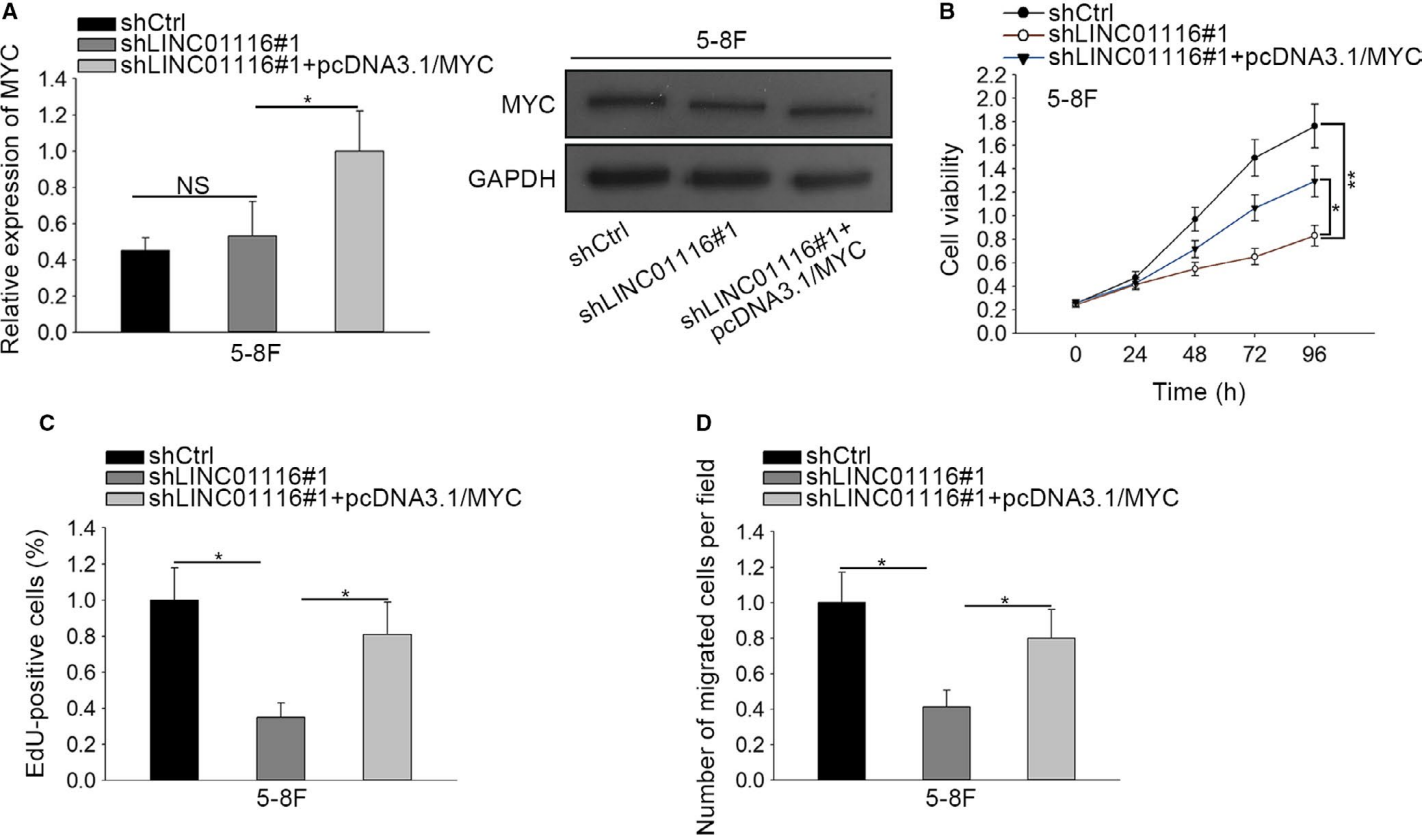
Overexpression of MYC rescued LINC01116 depletion‐restrained proliferation and migration in NPC cells. A, The expression of MYC at both mRNA and protein levels in indicated cells was detected via qRT‐PCR and western blot. B‐D, The viability, proliferation, and migration in 5‐8F cells under different conditions were respectively evaluated through CCK‐8 (B), EdU (C), and transwell assays (D). *P < .05, **P < .01

In order to confirm whether MYC was involved in LINC01116‐contributed NPC development, the rescue assays were performed in 5‐8F cells. As displayed in Figure 4A, both the mRNA and protein levels of MYC were dramatically prompted in LINC01116‐silenced 5‐8F cells in the context of MYC upregulation. Subsequently, we discovered that enforced expression of MYC resulted in a remarkable recovery on LINC01116 knockdown‐suppressed viability of 5‐8F cells (Figure 4B). Accordantly, the confined cell proliferation was also normalized in LINC01116‐inhibited NPC cells responding to the cotransfection of pcDNA3.1/MYC (Figure 4C). Similarly, MYC upregulation also counteracted the suppression of LINC01116 silence on the migration process of 5‐8F cells (Figure 4D). Therefore, we came to a conclusion that LINC01116 aggravates NPC progression through upregulating MYC.

### 3.5 LINC01116 is a direct target of c‐MYC

**Figure 5:**
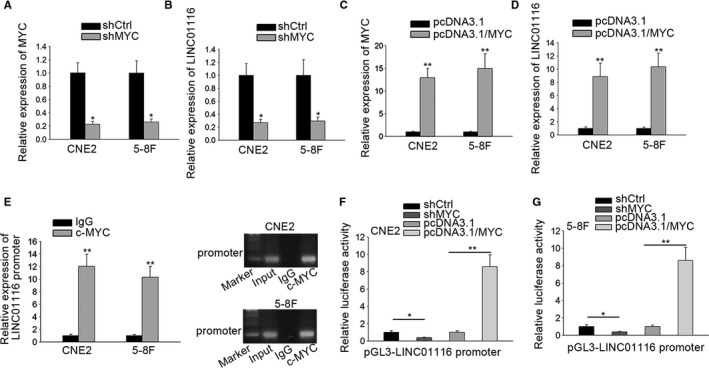
MYC activated LINC01116 at transcriptional level in NPC. A‐D, The expression of MYC or LINC01116 in indicated CNE2 and 5‐8F cells was tested by qRT‐PCR. E, ChIP assay and AGE analysis verified the binding of MYC protein to LINC01116 promoter. F,G, Luciferase reporter assay confirmed that LINC01116 was transcriptionally activated by MYC in both CNE2 and 5‐8F cells. *P < .05, **P < .01

In the meantime, the UCSC suggested that LINC01116 might be regulated by c‐MYC at transcriptional level. In this basis, we wondered whether MYC could affect LINC01116 expression in turn. As a consequence, we illustrated that LINC01116 was positively regulated by c‐MYC in NPC cells, evidenced by sharply reduced LINC01116 level in face of MYC silence but considerably stimulated LINC01116 level under MYC overexpression (Figure 5A‐D). Furtherly, the results of ChIP assay plus AGE analysis showed that LINC01116 promoter was noticeably harvested by anti‐c‐MYC in both CNE2 and 5‐8F cells (Figure 5E), indicating the direct binding affinity of c‐MYC and LINC01116 promoter in NPC cells. Additionally, we revealed that the luciferase activity of LINC01116 promoter in CNE2 cells was significantly abrogated by MYC inhibition but was robustly encouraged under MYC upregulation (Figure 5F), whereas a similar phenomenon was also observed in 5‐8F cells (Figure 5G). By and large, we demonstrated that LINC01116 is transactivated by c‐MYC in NPC.

## 4 DISCUSSION

In recent years, reports have revealed that aberrant expression of lncRNAs can modulate cell proliferation and migration in various tumors.^20^ The implication of lncRNAs in NPC, a common malignancy with high death rate, has also been demonstrated.^21^ For instance, DANCR plays a metastasis‐promoting role in NPC.^22^ ANRIL activated by SOX2 facilitates cell growth in NPC.^23^ NEAT1 affects NPC cell migration through targeting miR‐101‐3p.^24^ In this study, we investigated a novel lncRNA LINC01116 in the development of NPC. LINC01116 was previously uncovered to be upregulated and act as an oncogene in several cancers, including osteosarcoma,^11^ prostate cancer.^12^ Consistent with these findings, we proved in this study that LINC01116 was highly expressed in NPC cells and its suppression hindered NPC cell proliferation and migration.

It is known that the role of lncRNAs is closely associated with their localization in cells.^13^ For example, the cytoplasmic NF‐κB interacting long noncoding RNA (NKILA) inhibits metastasis in breast cancer.^25^ Zhang et al revealed that CCAT1 could serve as a scaffold in nucleus and as a molecular decoy for miR‐7 in cytoplasm in esophageal squamous cell carcinoma cells.^26^ Currently, we figured out that LINC01116 was predominantly distributed in the cytoplasm of NPC cells.

Considering that cytoplasm‐distributed lncRNAs can regulate the expression and/or localization of RNAs and/or proteins via directly interacting with them,^27^, ^28^ we then probed certain RNA or protein which interacted with LINC01116 in NPC. Previously, Su et al discovered that NPCCAT1 enhances YY1 activity in NPC cells through interacting with YY1 mRNA.^14^ Herein, we uncovered that LINC01116 in the cytoplasm could interact with MYC mRNA in a similar way. More intriguingly, it was unveiled that LINC01116 accelerated MYC translation through its interaction with 5’UTR of MYC mRNA via the complementary sequences, similar to previous findings that lncRNAs can enhance protein translation via interacting with their antisense mRNAs.^15^, ^29^ C‐MYC is a protumor transcription factor that triggers unlimited cell growth and metastasis in cancer through activating or repressing the transcription of its target genes.^30^, ^31^ In this study, we discovered that LINC01116 promoted NPC tumorigenesis through elevating c‐MYC protein so as to enhance c‐MYC transcriptional activity and upregulate c‐MYC targets like CDK4,^17^ CCND2, 18 and BMI1.^19^ Meanwhile, it was also identified that LINC01116 was transcriptionally activated by c‐MYC in NPC cells.

On the whole, our study unveiled a LINC01116‐MYC feedback loop in promoting NPC progression, thus indicating LINC01116 as a new biology marker for the treatment of patients with NPC. Nevertheless, the clinical value of LINC01116 in NPC therapy is still poor now, and more studies regarding the findings in this study are badly needed.

## CONFLICT OF INTEREST

None.

## AUTHOR CONTRIBUTIONS

Haijie Xing, Hongxia Sun and Weiluo Du: project administration, investigation, article writing and review, experiment, data curation, figures. All authors gave valuable suggestions and all authors contribute equally.

## Data Availability

Research Data are not shared.
