# Supplementary figures and images for: LINC01116 accelerates nasopharyngeal carcinoma progression based on its enhancement on MYC transcription activity
Source: Cancer Med. 2019 Nov 8;9(1):269–77. doi: 10.1002/cam4.2624 (PMC6943083; doi:10.1002/cam4.2624)

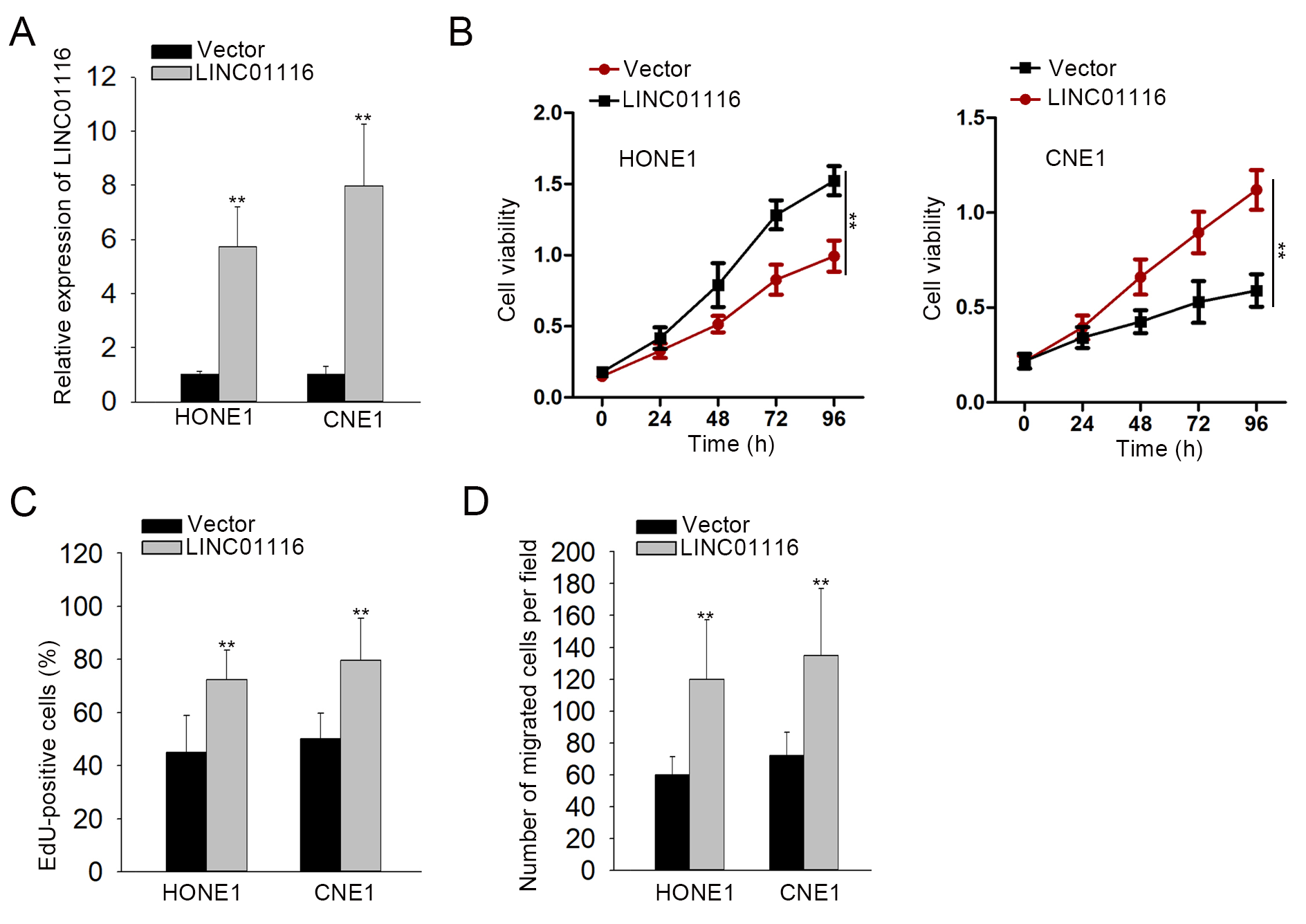

Supplement: Supplementary file 1 [file CAM4-9-269-s001.tif]

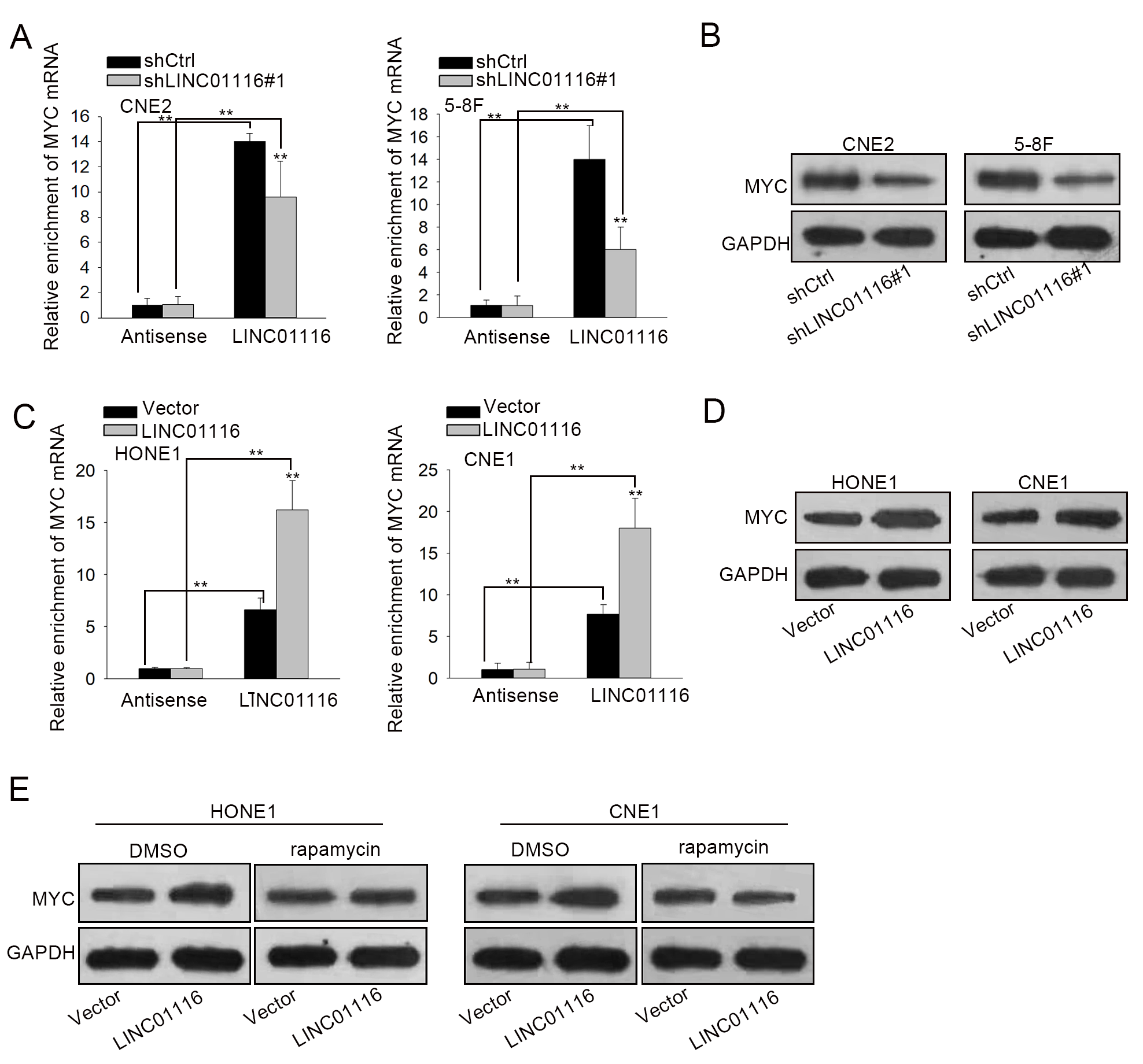

Supplement: Supplementary file 2 [file CAM4-9-269-s002.tif]
